# Myalgische Enzephalomyelitis/chronisches Fatigue-Syndrom: eine Übersicht zur aktuellen Evidenz

**DOI:** 10.1007/s00115-022-01431-x

**Published:** 2023-01-25

**Authors:** Birgit Ludwig, Elisabeth Olbert, Karin Trimmel, Stefan Seidel, Paulus S. Rommer, Christian Müller, Walter Struhal, Thomas Berger

**Affiliations:** 1 Universitätsklinik für Neurologie, Medizinische Universität Wien, Währinger Gürtel 18–20, 1090 Wien, Österreich; 2 Universitätsklinik für Neurologie, Karl Landsteiner Privatuniversität für Gesundheitswissenschaften, Tulln, Österreich

**Keywords:** Evidenz, Post-COVID, Psychosomatik, Randomisiert-kontrollerte Studien, POTS, Evidence, Post-COVID, Psychosomatic, Randomized-controlled trials, POTS

## Abstract

In den vergangenen 5 Jahren hat sowohl das mediale als auch das wissenschaftliche Interesse an der Erkrankung myalgische Enzephalomyelitis/„chronic fatigue syndrome“ (ME/CFS) signifikant zugenommen; nicht zuletzt auch durch die klinisch ähnliche Manifestation im Rahmen von Long- oder Post-COVID. In dieser Übersichtsarbeit diskutieren wir die klinische Diagnosestellung und therapeutische Studien zu ME/CFS sowie die Gemeinsamkeiten oder Unterschiede zu Long‑/Post-COVID. Bisher liegen weder pathophysiologisch eindeutig kausale noch therapeutisch evidenzbasierte Ergebnisse in der langjährigen wissenschaftlichen Forschung zu ME/CFS vor. Nicht zuletzt aufgrund der relevanten psychiatrischen Komorbiditätsrate beim ME/CFS ist nach der aktuellen Datenlage eine psychosomatische Ätiologie der Erkrankung zu diskutieren. Des Weiteren könnte sich eine genauere und sichere Diagnosestellung anhand strikterer Diagnosekriterien auf die weitere Forschung und vor allem hinsichtlich Therapien positiv auswirken.

## Abstract (en)

Over the past 5 years both media and scientific interest has surged regarding the disorder myalgic encephalomyelitis and chronic fatigue syndrome (ME/CFS), not least because of the clinically similar manifestation in long COVID or post-COVID. In this review we discuss the process of clinical diagnosis and randomized controlled therapeutic studies on ME/CFS, and the similarities or differences to long COVID and post-COVID. So far, neither clear pathophysiologically causal nor therapeutic evidence-based results on ME/CFS have been identified in the many years of scientific research. Given the evident psychiatric comorbidity rates in patients with a diagnosis of ME/CFS, a psychosomatic etiology of this syndrome should be considered. Furthermore, a precise and reliable diagnostic classification based on stricter criteria would benefit both pathophysiological and therapeutic research.

Myalgische Enzephalomyelitis/„chronic fatigue syndrome“ (ME/CFS) ist ein seit Jahrzehnten kontrovers diskutiertes Thema. In den vergangenen 5 Jahren ist es von wissenschaftlicher als auch von medialer Seite zu einem zunehmenden Interesse an ME/CFS gekommen. In der Wissenschaftsdatenbank „PubMed“ finden sich für den Suchbegriff „ME CFS“ 2017 192 Artikel, 2021 schon 314 neue Publikationen. Zusätzlich klagen mehr und mehr Menschen über ME/CFS-ähnliche Symptome nach einer Infektion mit SARS-CoV‑2. Insofern ist eine zeitgemäße Übersicht zur bestehenden Evidenzlage bei ME/CFS angebracht.

## Historische Betrachtungen

Der Terminus „myalgische Enzephalomyelitis“ dürfte im Zusammenhang mit ungeklärten Infektionswellen zwischen 1948 und 1955, die damals als „poliomyelitis-simulierend“ bezeichnet wurden und an den unterschiedlichsten Orten der Welt – von Island über die USA bis nach Australien – auftraten [28], aufgekommen sein. Insgesamt sind 15 Infektionswellen aufgezeichnet, die wahrscheinlich am besten dokumentierte ereignete sich im Royal Free Hospital in London [43]. Der Großteil der Betroffenen waren Spitalsangestellte, die über Kopf- und Halsschmerzen, geschwollene Lymphknoten, Myalgie, Schwäche sowie erhöhte Temperatur klagten. Die Bezeichnung als „Enzephalomyelitis“ rührt von der damaligen klinischen Beobachtung, dass 74 % der Betroffenen auch neuropsychiatrische Beschwerden (Hypersomnie, Panikattacken, unkontrollierbares Weinen, Beteiligung der Hirnnerven) zeigten. Gemein ist all diesen Schilderungen ein protrahierter Verlauf mit Fatigue, schlaffer Muskelschwäche und psychiatrischen Symptomen monatelang nach Beginn der Erkrankung. Moderne bildgebende Verfahren gab es damals noch nicht, aber die durchgeführten Liquoruntersuchungen ergaben weder Hinweise auf einen entzündlichen Prozess, noch konnte ein kausales infektiöses Agens identifiziert werden. Insgesamt sind die Schilderungen hinsichtlich ihrer kausalen Zusammenhänge mit Vorbehalt zu betrachten, aber jedenfalls medizinhistorisch interessant. Die Bezeichnung „chronic fatigue syndrome“ wurde erstmals 1988 zur Beschreibung ME-ähnlicher Symptome in Nevada, USA, verwendet [31].

Trotz Anwendung moderner molekularbiologischer und bildgebender Methoden ist die Ätiologie und Pathogenese von ME/CFS nach rund 70 Jahren weiterhin enigmatisch. Weder in der Liquordiagnostik, noch morphologisch (Magnetresonanztomographie [MRT], aber auch neuropathologisch) konnte bislang ein entzündliches Korrelat, wie es die Bezeichnung „Enzephalomyelitis“ suggeriert, identifiziert werden [52]. Die Beschreibung des zu erwartenden Krankheitsverlaufs in der Nomenklatur ist zu hinterfragen, die Studienlage hierzu ist unklar und Beobachtungen von Langzeitverläufen sind durch die in Änderung begriffenen Diagnosekriterien nicht einheitlich zu bewerten. So konnte in einer Publikation gezeigt werden, dass 25 Jahre nach Erstdiagnose 20 der 25 initial diagnostizierten Patient:innen zum Zeitpunkt der Begutachtung die Diagnosekriterien von Fukuda nicht erfüllten und 15 der 20 remittierten Patient:innen wieder fähig waren, zu arbeiten [13]. Eine Revision der Bezeichnung wäre daher aus heutiger schlussfolgernder Sicht notwendig, um zukünftig weitere terminologische Inkongruenzen und Verwirrungen – auch in Abgrenzung zu tatsächlich klar definierten (Enzephalo‑)Myelitiden – zu vermeiden. Wir plädieren, solange keine Evidenz zur Pathogenese besteht, für eine deskriptive Bezeichnung der persistierenden Erschöpfung als „chronic fatigue syndrome“. Bei bislang fehlendem Nachweis einer tatsächlichen Enzephalomyelitis (z. B. mittels MRT-Bildgebung oder neuropathologischem Befund) ist der Begriff „Enzephalomyelitis“ rein suggestiv und daher aus Sicht der Autor:innen nicht gerechtfertigt.

## Klinische Betrachtungen

Die US Centers for Disease Control and Prevention (CDC) definieren ME/CFS als komplexe, chronische und beeinträchtigende Multisystemerkrankung mit multiplen pathophysiologischen Veränderungen. Die österreichische ME/CFS-Hilfe schließt sich dieser Definition an (https://cfs-hilfe.at/wp-content/uploads/2022_01-Factsheet-kurz.pdf zuletzt eingesehen am 04.04.2022). Trotz jahrzehntelanger Forschung sind die von der CDC beschriebenen pathophysiologischen Veränderungen nicht in einer Weise reproduzierbar bzw. von „pathophysiologischen Veränderungen“ bei Gesunden abgrenzbar, dass es zu der Entwicklung und Anerkennung eines Biomarkers gekommen wäre, – ein Umstand den die Erkrankung mit psychiatrischen Erkrankungen teilt (abgesehen von F00–F09 Diagnosen). Auf die – insbesondere von Selbsthilfegruppen – gewünschte Abgrenzung zu psychiatrischen und psychosomatischen Erkrankungen wird noch eigens an späterer Stelle eingegangen.

Die rezentesten Diagnosekriterien wurden vom US Institute of Medicine [19] veröffentlicht (Infobox 1). Methodische Limitationen dieser Kriterien betreffen das Einbeziehen subjektiver Beschreibungen (Dauer der chronischen Erschöpfung, Intoleranz/Verschlechterung bei körperlicher Anstrengung, nichterholsamer Schlaf) und objektiv messbarer Beeinträchtigungen (kognitive bzw. orthostatische Dysfunktion). Dabei sind die Kriterien bzw. empfohlenen Testmodalitäten zu den kognitiven Beeinträchtigungen unklar, obwohl Defizite in Aufmerksamkeit, Gedächtnis und Reaktionszeit in neuropsychologischen Untersuchungen [20] gezeigt wurden. Darüber hinaus ist die orthostatische Intoleranz (OI) ein Überbegriff für unterschiedliche Syndrome des autonomen Nervensystems (beispielsweise orthostatische Hypotonie, Reflexsynkope oder posturales orthostatisches Tachykardiesyndrom [10]). Das posturale orthostatische Tachykardiesyndrom (POTS) wird als Anstieg der Herzfrequenz um über 30 Schläge pro Minute, ohne begleitende Hypotension, mit Symptomen wie „Schwindel“, „brain fog“, Palpitationen, Tremor, generalisierte Schwäche, Verschwommensehen, Belastungsintoleranz oder Fatigue in einem Stehversuch von 10 min definiert [66]. Die Diagnose kann, bei seit 3 bis 6 Monaten anhaltenden Symptomen, anhand dieser Kriterien im Rahmen einer Kipptischuntersuchung, aber auch niederschwellig im Rahmen eines standardisierten medizinischen Stehversuchs gestellt werden [11]. Im Rahmen einer kleinen Studie an POTS-Patient:innen erfüllten 64 % die klinischen Kriterien eines CFS und 93 % der Patient:innen berichteten Fatigue-Symptome, orthostatische Intoleranz, Palpitationen, Schwindel, Tagesmüdigkeit und gastrointestinale Störungen [51]. Eine rezente Studie vermutet Angstkonditionierung für die aufrechte Haltung als Pathophysiologie von POTS. Patient:innen mit POTS hatten einen höheren antizipatorischen Herzratenanstieg, schon bevor sie in die 70°-Stellung des Kipptisches gebracht wurden. Somatische Vigilanz und Katecholaminanstieg während der aufrechten Haltung waren Prädiktoren für den Herzratenanstieg und die Hyperventilation. Diese Ergebnisse wurden als Hinweise für einen psychogenen bzw. funktionellen Ursprung des Syndroms gedeutet [49]. Kritisch zu hinterfragen bei dieser Studie sind folgende Punkte: erstens stellt sich die Frage, ob die in den 30 s auftretende Tachykardie zwischen akustischer Warnung und tatsächlichem Aufrichten eine hinreichende Erklärung sind für eine tatsächlich 10 min anhaltende erhöhte Herzrate während des Kippens, zweitens sind 28 Patient:innen als Stichprobe für generelle Aussagen ungenügend und es gibt (noch) keine replizierten Daten diesbezüglich. Vergleichbar, wenn auch anders in der Reizsetzung, ist eine ältere Studie, die versuchte Patient:innen nach einer Messung mit Unterdruckhose mit einem Stroop-Test mental zu stressen und hier fanden sich keine Unterschiede zwischen POTS-Patient:innen und gesunden Kontrollen (allerdings in einer noch kleineren Stichprobe; [42]).

In dem rezentesten systematischen Verzeichnis der Internationalen statistischen Klassifikation der Krankheiten und verwandter Gesundheitsprobleme (ICD-11) findet sich die Diagnose postvirales Fatigue-Syndrom (8E49) unter den neurologischen Erkrankungen und inkludiert das chronisches Fatigue-Syndrom und myalgische Enzephalomyelitis. Diese Diagnose kann nicht gestellt werden, sollte es sich um Fatigue (MG22) handeln, die wiederum Lethargie und eine generelle physische Verschlechterung inkludiert [78]. Die zahlreichen Differenzialdiagnosen (neurologischer, psychiatrischer und internistischer Genese), die bei Fatigue (MG22) als exkludierende Diagnosen angeführt werden, werden bei postviralem Fatigue-Syndrom interessanterweise nicht angeführt.

Die aktuellen National Institute for Health and Care Excellence(NICE)-Guidelines [1] zu Diagnose und Management von ME/CFS empfehlen ME/CFS bei Erfüllung der o. g. Kriterien (allerdings schon nach 3 Monaten) zu suspizieren, aber die Diagnosestellung ME/CFS-Expert:innen zu überlassen [68]. Im Gegensatz zur früheren NICE-Veröffentlichung [6] sind nun in den neuen Richtlinien keine Interventionen mit kognitiver Verhaltenstherapie („cognitive behavioral therapy“, CBT) und stufenweiser Aktivierung („graded exercise therapy“, GET) mehr empfohlen. Obwohl sogar ein Cochrane-Review eine Verbesserung der Fatigue durch CBT und GET zeigte [37], bestand aber seitens der Patient:innen der Vorwurf, dass diese Behandlungen nachteilig wären [67]. Trotz der Übernahme dieser Empfehlung durch die Mayo Clinic und der CDC [8] werden diese auch kritisch gesehen [22].

Die pathophysiologischen Grundlagen des ME/CFS werden kontrovers diskutiert. Die bis dato unbestätigte Hypothese hierzu geht von einer überschießenden Autoimmunantwort nach einer (oft stummen) viralen Infektion aus [46]. Insbesondere die humanen Herpesviren wie Epstein-Barr-Virus bzw. deren Reaktivierung wurden oft verdächtigt, am Anfang der ME/CFS-Erkrankung zu stehen [5] und eine chronisch inflammatorische Antwort zu generieren. Wie bei allen pathophysiologischen Hypothesen zu ME/CFS finden sich einige Studien mit immunologischen Auffälligkeiten (beispielhaft: reduzierte zytotoxische NK-Aktivität, erhöhte Interleukin-10-Werte), die aber in nachfolgenden Studien nicht repliziert werden konnten [57]. Im Serum von ME/CFS-Patient:innen wurden gegenüber gesunden Kontrollen höhere G‑Protein-gekoppelte Rezeptorautoantikörper (M3-, M4- und β2-Autoantikörper), die eine Dysregulation nachgeschalteter anticholinerger und adrenerger Signalwege bedingen, gefunden. Der Unterschied in den oben genannten Autoantikörpern war signifikant (im Gegensatz zu den ebenfalls getesteten M1-, M2-, M5- und β1-Autoantikörpern), aber für die Verwendung als diagnostischen Marker nicht umsetzbar, da zu viele gesunde Kontrollen ebenfalls sehr hohe Werte im ELISA zeigten [39]. Andere Erklärungsmodelle spekulieren über metabolische, mitochondriale, hämodynamische und selbst bildgebende Unterschiede zwischen ME/CFS-Patient:innen gegenüber Gesunden, wobei die tatsächliche Evidenz aufgrund mangelnder Sensitivität und Spezifität dieser Befunde und dem Fehlen einer biologischen nachweisbaren Methode limitiert ist [36]. Ebenso konnte die Überlegung einer autoimmunen Genese nicht bewiesen werden. Bei den verschiedenen Antikörpern (z. B. M3-, M4- und β2-Autoantikörper), die bei ME/CFS getestet wurden, konnte keine klinische Relevanz gezeigt werden. Für weitere Überlegungen verweisen wir diesbezüglich auf eine rezente Übersichtsarbeit von Muller et al. [46].

## ME/CFS und neurologische Beschwerden nach Infektion mit SARS-CoV 2 im Vergleich

Die Diskussion rund um ME/CFS hat jetzt nicht zuletzt aufgrund der rezent aufgetretenen Post-COVID/Long-COVID-Symptome wieder an Aktualität gewonnen. Die S1-Guidelines der Arbeitsgemeinschaft der Wissenschaftlichen Medizinischen Fachgesellschaften e. V. (AWMF) [35] sowie die NICE-Guidelines [59] orientieren sich an folgender Definition: Bei anhaltenden Beschwerden jenseits einer Zeitspanne von 4 Wochen ab Infektion handelt es sich um Long-COVID oder postakute Folgen von COVID-19. Beim Auftreten innerhalb von 12 Wochen nach Infektion und Persistenz von mindestens 2 Monaten werden diese Beschwerden laut World Health Organization (WHO) als Post-COVID-19-Syndrom bezeichnet [77]. Fraglich bleibt, ob dieser Syndrombegriff der Vielzahl und auch der Variabilität der Symptome und auch potenziellen Differenzialdiagnosen gerecht wird [4]. Die epidemiologischen Zahlen klaffen weit auseinander: In einer App-basierten Studie berichteten von 4182 SARS-CoV-2-Infizierten lediglich 13,3 % von anhaltender Fatigue, Kopfschmerzen, Dyspnoe und Geruchsverlust 28 Tage nach Infektion und nach 12 Wochen nur mehr 2,3 % [64]. In einer anderen Studie mit 938 Teilnehmer:innen antworteten 46 %, dass sie nach 1,5 bis 6 Monaten (Mittelwert 117,5 Tage) nach Infektion noch immer unter Fatigue leiden würden [61]. Vermeintliche Gemeinsamkeiten zwischen Post-COVID- und ME/CFS-Symptomen sind zum einen die Fatigue bzw. die Belastungsintoleranz und die subjektiven kognitiven Beeinträchtigungen, aber weder Kopfschmerzen noch Geruchsverlust sind in den Diagnosekriterien von ME/CFS enthalten. Inwieweit Patient:innen nach COVID-Infektion häufiger orthostatische Dysfunktion als die Normalbevölkerung entwickeln, ist, trotz mehrere Berichte, noch ungeklärt [14]. Die Pathophysiologie der neurologischen Post-COVID-Symptome ist bislang ungeklärt, unter anderem wird diskutiert, ob SARS-CoV‑2 ein neurotropes Virus sei. Hinweise darauf wären einzelne histologische Fallberichte, die das Virus in neuronalen Endothelzellen nachweisen [53], allerdings wurde nie ein aktives sich tatsächlich replizierendes Virus gefunden. In einer prospektiven Studie wurden 40 Patient:innen mit neurologischen Symptomen während und nach der SARS-CoV-2-Infektion lumbalpunktiert. Im Liquor fanden sich keine Hinweise auf eine aktive Infektion des Zentralnervensystems, bei 5 Patient:innen konnte das Virus per PCR nachgewiesen werden, allerdings mit sehr niedrigen Werten [58]. Neuropsychologische Studien zeigen eine Diskrepanz zwischen den wahrgenommenen kognitiven Einschränkungen und den Resultaten in den normierten neuropsychologischen Tests, aber Assoziationen zwischen hohen Werten in Angst- und Depressionsfragebögen und grenzwertig unterdurchschnittlichen Ergebnissen in den kognitiven Testungen [69, 75, 76]. Werden die von Patient:innen geschilderten Beschwerden strukturiert aufgearbeitet, so lassen sich bei der Mehrheit keine neurologisch objektivierbaren Symptome finden (85,5 %) und bei einer geringeren Prozentzahl an Patient:innen tatsächlich neurologische Symptome, die aber etablierten neurologischen Diagnosen zuordenbar ist [80]. In dieser prospektiven Kohortenstudie von Fleischer et al. konnte auch ein signifikanter Zusammenhang zwischen Fatigue und Konzentrationsschwierigkeiten mit vorbekannten psychiatrischen Erkrankungen dargestellt werden [80]. Während mittlerweile fast sämtliche ME/CFS-Richtlinien vermeiden, psychische, psychosomatische und psychiatrische Aspekte zu erwähnen, findet sich in den Empfehlungen der S1 ein Kapitel mit psychischen Post-COVID-Komponenten und in den allgemeinmedizinischen Empfehlungen die Aufforderung, eine psychosomatische Grundversorgung anzubieten [35]. Longitudinale Studien mit höherer Fallzahl an Proband:innen, normierten neuropsychologischen Testungen und zerebralen MRT-Kontrollen werden notwendig sein, um systematisch und evidenzbasiert die von den Patient:innen geschilderten Beschwerden schlussendlich korrekt zu definieren und klassifizieren.

## Psychosomatische Sichtweise

Während die Debatte, ob ME/CFS eine primär physische oder psychische Erkrankung ist, fast vollkommen aus dem wissenschaftlichen Diskurs verschwunden ist, betonen Selbsthilfegruppen, dass ME/CFS mit zumindest nicht unerheblicher psychischer (Mit‑)Komponente (fehl‑)wahrgenommen wird. Internationale Gremien (ICD-10, NICE-Guidelines [68], Mayo Clinic [8]) haben sich klar positioniert, lassen aber eine breitere patient:innenspezifische Herangehensweise nicht zu. Eine somatoforme Störung wird diagnostiziert, wenn physische Symptome medizinisch nicht oder nicht in ihrer präsentierten Intensität erklärbar sind. Die Positionierung von ME/CFS als eigenständige Diagnose im ICD-10 und die ungenauen, einzig auf Anamnese basierenden Diagnosekriterien machen es praktisch unmöglich, bei chronischer Fatigue eine somatoforme Störung statt ME/CFS zu diagnostizieren.

In einer deutschen Arbeit untersuchen Forscher:innen die Prävalenz chronischer Fatigue (operationalisiert anhand eines Cut-off-Scores für Fatigue und > 6 Monate andauernd – damit zwar ähnlich, aber nicht vollkommen den ME/CFS-Kriterien entsprechend) und somatoformer Störungen (operationalisiert anhand von zwei Fragebögen) in der Allgemeinbevölkerung. Die chronische Fatigue und somatoforme Störungen traten in einer Überlappung von 72 % auf [41]. Während in den letzten beiden Jahrzehnten ein Meinungswandel zu beobachten war, wurde ME/CFS um und vor dem Jahr 2000 in Übersichtsarbeiten oft unter somatoformen Störungen eingereiht. Die starke Überlappung verschiedener medizinisch unerklärter physischer Beschwerden ist gut dokumentiert: Die spezifischen Statistiken und Zusammenhänge variieren, heben sich jedoch signifikant von der Feldprävalenz der einzelnen Erkrankungen (unter anderem ME/CFS, Reizdarmsyndrom, Fibromyalgie und temporomandibuläre Dysfunktion) ab [2]. 1970, ca. 15 Jahre nach dem Ausbruch im Royal Free Hospital, argumentierten zwei Psychiater nach einer retrospektiven Analyse der Aufzeichnungen der Patient:innen, dass es sich dabei um eine „Massenhysterie“ gehandelt haben muss [44]. In ihrer Argumentation beziehen sie sich vor allem darauf, dass in den Untersuchungen weder Biomarker noch Erregerquellen gefunden worden seien, dass es sich bei den Patient:innen vor allem um Frauen handle und schließlich, dass die Beschwerden der Patient:innen nicht in Relation zu dem niedrigen Fieber stünden. Sie schlagen in weiterer Folge vor, die damalige Erkrankung und auch ME/CFS in „Myalgia nervosa“ umzubenennen und unter den funktionellen Erkrankungen einzureihen [43].

Grundsätzlich wird es immer psychoanalytische Theorien zu Erkrankungen mit unklarer Genese (beispielhaft: Fibromyalgie und Reizdarmsyndrom) geben [55]. Aus psychodynamischer Sicht ist die von Freud untersuchte und behandelte Neurasthenie in diesem Sinn von Bedeutung. Insofern wird ME/CFS von Vertreter:innen der Psychoanalyse heute dem psychosomatischen Formenkreis zugeordnet [12].

In einem Fallbericht mit zwei ME/CFS-Patient:innen von 1994 schlussfolgerten die Analytiker:innen nach 18 Monaten Therapie in einem hochfrequenten Setting (mit 2 bis 3 Sitzungen pro Woche), dass die Schwierigkeiten aus einem Mangel an Internalisierung selbstregulierender Strukturen in einer frühen Beziehung zur Bezugsperson entstammen [65]. Den Ausbruch der Erkrankung im Erwachsenenalter bringen die Autor:innen mit einem Bruch einer dafür kompensierenden Beziehung in Zusammenhang. Des Weiteren bemerkten sie, dass die beiden Patient:innen im Verlauf der Behandlung Schwierigkeiten hatten, Affekte zu identifizieren, zu benennen und zu regulieren, und über einen langen Zeitraum der Behandlung körperliche Beschwerden benutzt wurden, um Affekte auszudrücken [65]. In einer rezent erschienenen Metaanalyse mit 17 randomisiert-kontrollierten Studien zu psychodynamischen Kurzzeittherapie bei funktionalen somatischen Symptomen fanden die Autor:innen eine mäßige bis starke Besserung der somatischen Symptome; allerdings gibt es keine Ergebnisse spezifisch für ME/CFS [3]. Eine gute Datenlage existiert bezüglich der psychiatrischen Komorbiditäten bei ME/CFS-Patient:innen: die Rate liegt zwischen ca. 45 % [40, 48] und 70 % [47, 71]. Von Seite der Patient:innen wird hingegen argumentiert, dass psychiatrische Komorbiditäten als Reaktion auf die belastende Erkrankung entstünden. Dieser Hypothese setzt eine Arbeit aus London überraschende Daten aus dem Geburtskohortenregister entgegen, indem sie die Hypothese aufstellten, dass ME/CFS-Patient:innen bereits vor dem Beginn der Symptome erhöhte Raten an psychiatrischen Erkrankungen hatten. Insbesondere Depression und Angst gingen den Fatigue-Symptomen voraus und auch der Schweregrad der psychiatrischen Erkrankung schien vorherzusagen, ob eine ME/CFS-Diagnose im Laufe des Lebens gestellt wird [30].

In 2 Studien der Universitäten Leuven und Antwerpen wurden verschiedene Kohorten (ME/CFS-Patient:innen, psychiatrische Patient:innen und gesunde Kontrollen) in Bezug auf Persönlichkeitsstörungen untersucht. Hierbei zeigte sich bei beiden Studien eine mit gesunden Kontrollen vergleichbare Rate an Persönlichkeitsstörungen in der Kohorte der ME/CFS-Patient:innen (ca. 13 bzw. 16 %) und eine signifikant höhere Rate an Persönlichkeitsstörungen in der Kohorte der psychiatrischen Patient:innen (> 50 %; [21, 34]). Zusammenfassend besteht, basierend auf der aktuellen Datenlage, eine offensichtliche Assoziation zwischen ME/CFS mit sowohl psychiatrischen Krankheitsbildern als auch somatoformen Störungen. Die Richtung dieser Assoziation ist noch nicht endgültig geklärt, wenn es auch Hinweise darauf gibt, dass psychiatrische Erkrankungen den ME/CFS-Symptomen bereits vorausgehen.

## Randomisiert-kontrollierte Studien zur Behandlung von ME/CFS

**Figure 1:**
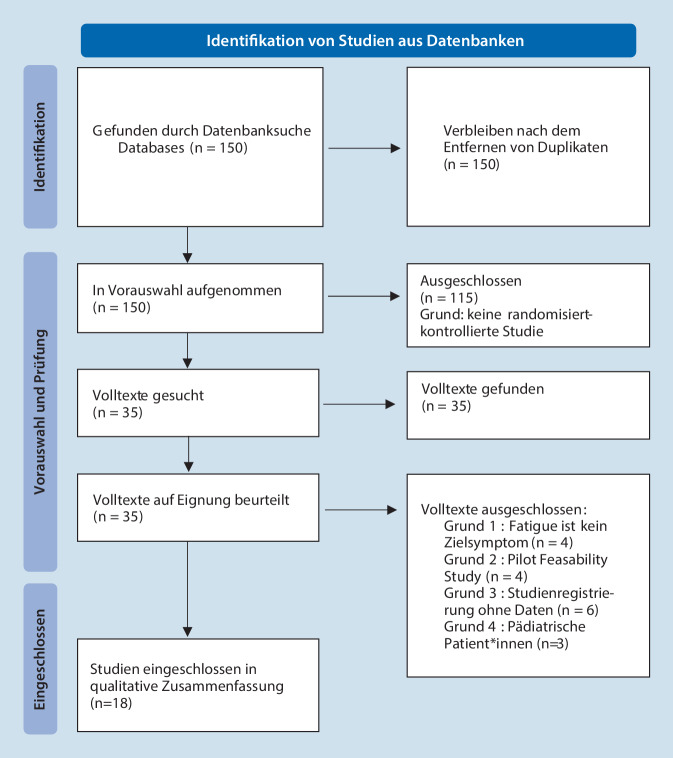

PubMed wurde systematisch und ohne zeitliche Beschränkung nach allen randomisiert-kontrollierten Studien zu ME/CFS durchsucht. Das Flussdiagramm (Abb. 1) gibt einen Überblick über die eingeschlossenen Studien.

In einer Phase-III-Studie aus den USA mit insgesamt 234 ME/CFS-Patient:innen zeigte sich im Vergleich zur Kontrollgruppe eine signifikante Verbesserung der körperlichen Leistungsfähigkeit in einem objektiven Test am Laufband, nachdem sie über einen Zeitraum von 40 Wochen 400 mg Rintatolimod (selektiver Agonist des Toll-like-Rezeptors 3) 2‑mal wöchentlich intravenös erhalten hatten [62].

Insgesamt gibt es nach einer Fallserie [24] 3 Studien [23, 25, 26] zur Behandlung von ME/CFS-Patient:innen mit dem monoklonalen B‑Zellen-depletierenden Antikörper Rituximab. In einer ersten doppelblinden Studie mit 30 Studienteilnehmer:innen zeigte sich 25 Wochen nach Behandlung eine signifikante Verbesserung in einem Selbsteinschätzungsfragebogen zu Fatigue-assoziierten Symptomen in der Kontrollgruppe [23]. Eine nachfolgende offene Studie mit 29 ME/CFS-Patient:innen zeigte bei 18 Patient:innen ein klinisches Ansprechen in einem Selbsteinschätzungsfragebogen [26]. Die letzte Studie derselben Autor:innen zeigte bei 151 Patient:innen hingegen keinen signifikanten Unterschied im Ansprechen (klinische Besserung der Fatigue-Symptome laut Selbsteinschätzungsfragebogen, Short form 36 Physical Form, gezählten Schritten und Fatigue Severity Scale) auf die Gabe von Rituximab (500 mg/m^2^ und dann 500 mg Fixdosis) insgesamt 6‑mal über einen Zeitraum von 12 Monaten verglichen mit der Placebogruppe [25].

Ähnlich widersprüchlich sind die Ergebnisse zu intravenösen Immunglobulinen (IVIg). Die erste doppelblinde, placebokontrollierte Studie aus dem Jahr 1990 vermerkte ein positives Ansprechen jener Patient:innen, die 2 g/kg IVIg erhalten hatten [38]. Limitationen dieser Studie waren die niedrige Fallzahl von 49 Patient:innen, zum anderen die Definition des Endpunktes des positiven Ansprechens. Zeitgleich führte eine andere Forschungsgruppe eine placebokontrollierte Studie mit monatlich verabreichten IVIg (1 g/kg) bei 28 Patient:innen durch und fand nach 6 Monaten keine klinische Verbesserung [54]. Auch die dritte placebokontrollierte Studie, in der 73 Patient:innen IVIg in verschiedener Dosierungen (0,5 g/kg, 1 g/kg, 2 g/kg) und 26 Patient:innen Placebo erhielten, fand keine signifikanten Unterschiede hinsichtlich der Leistungen im Karnofsky-Index bzw. den Ergebnissen der Fragebögen zu Lebensqualität, Aktivität und Stimmung zwischen Placebo und Wirkstoff [72].

Eine Studie mit Valganciclovir 900 mg 2‑mal täglich für 21 Tage, gefolgt von 90 mg einmal täglich für 6 Monate zeigte subjektive Verbesserungen über die Dauer von 9 Monaten in Fatigue-Fragebögen und kognitiver Leistungseinschätzung bei 30 ME/CFS-Patient:innen mit positiven Epstein-Barr virus (EBV)- und Humanes Herpesvirus 6(HHV-6)-IgG-Titern [45].

Weitere zahlreiche Studien mit Nahrungsmittelergänzungsmitteln bzw. bereits etablierten, pharmakologisch wirksamen, aber nebenwirkungsarmen Substanzen wurden publiziert. In einer spanischen Studie bekamen 50 ME/CFS-Patient:innen entweder 1 mg Melatonin und 10 mg Zink oder Placebo für bis zu 16 Wochen und zeigten keine signifikante Verbesserung im primären Endpunkt (Fatigue-Score) [17]. In einer anderen Studie wurde entweder 200 mg orales Koenzym Q10 und 10 mg Nikotinamid-Adenin-Dinukleotid-Hydrid (NADH) oder Placebo verabreicht. Nach 8 Wochen Behandlung zeigte sich weder in den Fatigue- noch in den Schlaffragebögen ein signifikanter Unterschied zwischen Verum und Placebo, einzig die subjektive Schlafeffizienz verbesserte sich in der Kontrollgruppe nach 8 Wochen Behandlung [16]. In einer japanischen doppelblinden Studie wurde über einen Zeitraum von 12 Wochen Ubiquinol-10 verabreicht. Eine signifikante Verbesserung zeigte sich für die objektiven Endpunkte (korrekte Antwortrate bei arithmetischen Aufgaben, nächtliches Erwachen und die Herzratenvariabilität), jedoch nicht für die subjektiven (Fatigue- und Depressionsfragebogen; [27]). Eine offene Studie aus den Niederlanden untersuchte den Effekt von Acetylcarnitin und Propionylcarnitin auf Fatigue (mittels Fragebogen) und Konzentrationsfähigkeit (mittels computerbasiertem Test). Die Fragestellung war, ob sich Acetylcarnitin positiv auf die Konzentration und Propionylcarnitin positiv auf die physische Leistungsfähigkeit auswirken würde, während eine Kombination beider Wirkstoffe beide Endpunkte positiv beeinflussen würde. Die Einzelwirkung nach 24 Wochen Therapie bestätigte sich, nicht jedoch die kombinierte Therapie [70]. Eine kleine Cross-over-Studie mit akutem Tryptophanabbau (experimentelle Methode, die mittels oral appliziertem L‑Tryptophan-defizientem Aminosäuregemisch die Vorstufe des Neurotransmitters Serotonin im Gehirn verringert) vs. Placebo brachte keinen signifikanten Unterschied in Fatigue, Stimmung und Konzentrationsfähigkeit [60]. Zwei weitere Studien stammen aus den Jahren 1990 und 1999: In der ersten Studie (doppelblind und placebokontrolliert) wurde 63 Patient:innen mit der Diagnose „postvirales Fatigue-Syndrom“ (Patient:innen, die seit mindestens einem Jahr, aber maximal seit 3 Jahren nach einer viralen Infektion mit Fieber unter belastungsabhängiger Fatigue, Konzentrationsschwierigkeiten und Muskelschmerzen litten) eine Mischung aus essenziellen Fettsäuren für 3 Monate verabreicht. Nach einem, aber auch nach 3 Monaten wurde ein signifikanter Vorteil der Behandlungsgruppe gezeigt, gemessen an Symptomen wie Fatigue, Schwindel, Konzentrationsfähigkeit anhand einer 3‑Punkte-Skala [9]. Weder die Anzahl der Proband:innen wäre ausreichend, um eine Aussage über die Effektivität der Behandlung zu treffen, noch ist das Messinstrument ausreichend reliabel. Die Studie wurde 9 Jahren später anhand der Oxford-Diagnosekriterien (die aktuell selbst in der Kritik stehen, zu überdiagnostizieren [7]) wiederholt. Dabei wurde dieselbe 3‑Punkte-Skala für körperliche Symptome, dieselbe Mischung aus essenziellen Fettsäuren in derselben Dosierung verwendet, aber die Ergebnisse konnten nicht repliziert werden [73].

Als Fazit zu den bislang publizierten pharmakologischen Studien bei ME/CFS ist die fehlende Replizierbarkeit der Ergebnisse zu konstatieren, was zumindest teilweise auf unterschiedliche Studiendesigns, unterschiedliche Outcomeparameter und nicht standardisierte Testungen zurückzuführen ist. Schlussendlich muss bei bislang ungenügender pathophysiologischer Grundlage und bei fehlendem Ansprechen auf Immuntherapien die Grundlage einer Autoimmunhypothese infrage gestellt werden.

### Nichtpharmakologische Studien

Es gibt zahlreiche Studien mit positiven Ergebnissen für CBT [29, 32, 33, 50], inklusive der viel zitierten und auch viel kritisierten PACE-Studie. Diese Studie mit 641 Studienteilnehmer:innen untersuchte über einen Zeitraum von 8 Jahren, wie sich Pacing (das Beachten individueller Belastungsgrenzen), stufenweise Aktivierung (GET) oder CBT randomisiert auf den Chalder Fatigue Score (Fragebogen) und die körperliche Funktionsfähigkeit im Short-Form-(36)-Gesundheitsfragebogen auswirken. Im Vergleich zur herkömmlichen Behandlung zeigten sich nach einem Jahr signifikante Verbesserungen in beiden Fragebögen für CBT und GET [74]. In einer interessanten Mediatoranalyse stellten sich angstvermeidende Überzeugungen als mediierender Faktor für das Ansprechen der Patient:innen auf GET und CBT heraus. Vereinfacht ausgedrückt, sprachen jene Patient:innen auf die Therapie (insbesondere GET) an, wenn sie ihre angstvermeidenden Überzeugungen bezüglich körperlicher Aktivierung ändern konnten [18]. Kritikpunkte an der PACE-Studie sind unter anderem eine unzureichende Definition von Genesung bei ME/CFS, eine unzureichende statistische Korrektur bei multiplem Testen und subjektive primäre Endpunkte bei fehlender Verblindung sowie suggestive Passagen in den Manualen [79]. Letztendlich ist die PACE-Studie aber die randomisiert-kontrollierte Studie mit den meisten Studienteilnehmer:innen mit longitudinalen Charakter bzw. Follow-up-Testungen über bis zu 8 Jahren und zeigt signifikante Verbesserungen in Fatigue und körperlicher Funktionsfähigkeit bei Therapie mit CBT und GET.

## Evidenzbasierte Therapie bei ME/CFS

Zwischen der wissenschaftlichen Studienlage und der praktisch-medikamentösen Behandlung von Patient:innen besteht eine deutliche Diskrepanz. Der Umstand, dass es keine FDA- bzw. EMA-approbierte medikamentöse Behandlung für ME/CFS gibt, verleitet ME/CFS-behandelnde Ärzt:innen oft zur Verschreibung von Off-label-Medikamenten. Einzig Rintatolimod ist in Argentinien für mäßige bis schwere Verläufe von ME/CFS zugelassen [63] und wurde aber von der amerikanischen Arzneimittelbehörde erstmals 2009 und dann nochmal 2012 begutachtet und aufgrund fehlender Daten zu Effektivität und Arzneimittelsicherheit nicht zugelassen [15]. Weitere Therapien [23, 26, 38] wurden von verschiedenen Behörden nicht zugelassen, weil diese Studien mit einer geringen Fallzahl mit nachfolgend größeren Kohorten nicht repliziert werden konnten. Trotzdem wird insbesondere IVIg weiterhin off-label verabreicht. Aktuell prüft eine Gruppe der Universität Bergen in Norwegen die Wirksamkeit des Immunsuppressivums Cyclophosphamid bei ME/CFS. Zuletzt zeigte sich in einer nichtplacebokontrollierten Phase-II-Studie ein Ansprechen bei mehr als der Hälfte der Patient:innen (operationalisiert durch Fragebögen und Messung der Schrittzahl der Patient:innen; [56]). Ob diese Ergebnisse einer placebokontrollierten Prüfung standhalten werden oder wie bei Rituximab und IVIg am Placebovergleich scheitern, wird sich zeigen.

## Konklusion

Zusammenfassend handelt es sich bei der Erkrankung ME/CFS, trotz langjähriger Bemühungen Ätiologie und Pathogenese zu identifizieren und objektiv paraklinische Parameter zu finden, weiterhin um eine rein klinische Diagnose, die auf Basis der Anamnese und Einschätzung der Patient:innen gestellt wird. Objektiv messbare kausalitätsbegründete und diagnostische Parameter für die Ärzt:innen fehlen. Ähnlich steht es um das „Post-COVID-Syndrom“ – wie bei ME/CFS fehlen häufig objektivierbare Beschwerden. Die Aufgabe für die behandelnden Ärzt:innen wäre es, (eindeutig behandelbare) Erkrankungen, die den Symptomen zugrunde liegen könnten, rigoros abzuklären (inkl. Orthostase, neuropsychologische Testung, Nervenleitgeschwindigkeit, MRT bei entsprechenden Auffälligkeiten) (siehe Tab. 1). Die Schlussfolgerung liegt nahe, dass strengere Diagnosekriterien aus „diplomatischen“ Gründen fehlen, wie auch in den letzten NICE-Guidelines versucht wird, den Wünschen von Patient:innen(‑Selbsthilfegruppen) gerecht zu werden. Dabei würden etwa definierte Grenzen bei den Resultaten einer neuropsychologischen Testung die Diagnosestellung erleichtern und auch Patient:innen die gewünschte Legitimation für die geschilderten kognitiven Beeinträchtigungen geben. Eine sichere und zweifelsfreie Diagnosestellung wirkt sich langfristig auch auf die Qualität und Homogenität der Forschungsergebnisse zur Ätiologie, Pathogenese und kausaler Therapie von ME/CFS aus. Möglicherweise aus Gründen der Stigmatisierung psychiatrischer Erkrankungen, ist die psychosomatische Perspektive (eine naheliegende bei fehlender Objektivierbarkeit der Symptome) fast komplett aus dem wissenschaftlichen und medialen Diskurs über ME/CFS verschwunden. Damit wird den Forscher:innen aber auch die Möglichkeit genommen, randomisiert-kontrollierte Studien zu Physiotherapie (bzw. GET) und Gesprächstherapien durchzuführen, und den Patient:innen die Möglichkeit genommen, Therapien diesbezüglich wahrzunehmen. Denn im Vordergrund und an erster Stelle stehen Menschen, die Beschwerden haben und denen zusteht, dass eine Abklärung, Diagnose und Therapie ihrer Beschwerden nach aktuellem und evidenzbasiertem medizinischem Wissensstand korrekt und nachhaltig erfolgt.

**Table 1:** 

Diagnostik	„Red flags“
Neurologischer Status	Fokal neurologische Defizite
Anamnese und internistischer Status	Lymphadenopathie, ungewollter Gewichtsverlust
Polysomnographie	Schlafapnoe, Restless-Legs-Syndrom, REM-Schlafstörung
Neuropsychologische Testung	Depressive Symptome, Angststörungen, demenztypische Befunde
Labor inkl. BZ, HbA1C, Schilddrüsenwerte, Muskelblut, AChR-Ak, ANA, Ferritin	Auffällige Laborbefunde
cMRT und spinales MRT (falls indiziert)	MS-typische Läsionen, neurodegenerative Prozesse, Raumforderungen, Vaskulitis
ACTH-Kurztest (internistisch)	Adrenerge Insuffizienz
Lumbalpunktion	Pleozytose, oligoklonale Banden

*BZ* Blutzucker, *cMRT* cerebrale Magnetresonanztomographie, *AChR-AK* Acetylcholinrezeptor-Antikörper, *ANA* Antinukleäre Antikörper, *ACTH* adrenocorticotropes Hormon, *REM* rapid eye movement, *MS* Multiple Sklerose

### Infobox Diagnosekriterien ME/CFS (Institute of Medicine, Clayton 2015 [19])

1. Erhebliche Reduktion oder Beeinträchtigung bei der Ausübung von Beruf, Bildung, sozialen oder persönlichen Aktivitäten, die mehr als 6 Monate anhält und von stark ausgeprägter Erschöpfung begleitet ist, nicht von Geburt an existiert, nicht das Resultat exzessiver Anstrengung ist und sich durch Ausruhen nicht merklich lindern lässt sowie
2. eine Zustandsverschlechterung nach Anstrengung (Post-Exertional-Malaise, PEM) und
3. ein nicht-erholsamer Schlaf.
4. Zusätzlich muss mindestens eine der folgenden Beschwerden vorliegen: kognitive Beeinträchtigung oder orthostatische Intoleranz.
